# Giant breast phyllodes tumor with silent thromboembolism: A case report

**DOI:** 10.1002/cnr2.1865

**Published:** 2023-08-14

**Authors:** Qinbo Wang, Lina Wei, Yuan Zhou, Yingjuan Ou, Haiyan Li

**Affiliations:** ^1^ Department of General Surgery (Breast Surgery) The Sixth Affiliated Hospital, Sun Yat‐Sen University Guangzhou China; ^2^ Department of Pharmacy The Sixth Affiliated Hospital, Sun Yat‐Sen University Guangzhou China; ^3^ Department of Graceland Medical Center The Sixth Affiliated Hospital, Sun Yat‐Sen University Guangzhou China; ^4^ Biomedical Innovation Center The Sixth Affiliated Hospital, Sun Yat‐sen University Guangzhou China; ^5^ Department of Gastroenterology The Sixth Affiliated Hospital, Sun Yat‐Sen University Guangzhou China

**Keywords:** computed tomography, phyllodes tumor, surgical therapy, venous thromboembolism

## Abstract

**Background:**

Phyllodes tumor (PT) is a solid fibroepithelial breast lesion with proliferation of stromal and epithelial elements, usually presents with a rapidly expanding feature. Venous thromboembolism (VTE) have been reported to increase the burden in terms of mortality and morbidity of malignant tumor, and associate with worsened survival. However, benign PTs with silent thromboembolism that have not yet been reported, we report an unusual case of massive benign PT that grew on the left side of the breast in a cauliflower‐shaped form and presented severe chronic blood loss and deep VTE.

**Case:**

A 37‐year‐old woman with uncontrolled pain presented a rapidly enlarging left breast mass, measuring approximately 30 × 20 × 15 cm^3^ that first started 25 years ago. color Doppler ultrasound showed a large mass lesion on the left breast and deep VTE, several enlarged lymph nodes in the left axilla and mediastinum, which presented a malignant character. However, the biopsies of the mass did not show evidence of malignancy and the pathology result was considered to be benign PT. The patient was treated with an inferior vena cava and anticoagulation, the operation was arranged according to the surgical procedure, the patient recovered very well after mastectomy.

**Conclusion:**

This case is unique in that the giant breast mass presented with malignant character, was eventually pathologically confirmed to be benign PT, and it's rare that the benign tumor accompanied with silent thromboembolism. This finding describes the atypia features of giant benign PT and reminds the surgeon to consider the factor of VTE and risk when encountering ulcerative benign breast tumor and avoid excessive treatment.

## 1 INTRODUCTION

The World Health Organization (WHO) classified phyllodes tumor (PT) into benign, borderline, and malignant grade categories based on a constellation of histological parameters.^1^ Despite various malignant PT and perioperative venous thromboembolism (VTE) have been previously described, but benign PT with silent thromboembolism have not yet been reported.^2^ Cancer‐associated thrombosis (CAT) including VTE is highly consequential for patients with cancer in which the tumor expresses procoagulant proteins to promote the formation of blood clots, and clotting proteins accelerate tumor growth, and both risk factors and treatment must be considered.^3^ We present a giant breast phyllode tumor with silent thromboembolism, biochemistry results showed severe anemia, and color Doppler ultrasound (CDU) showed extensive deep VTE; we also summarize clinical characteristics, differential diagnosis, and treatment of supergiant benign PT (maximum diameter was 30 cm).

## 2 CASE PRESENTATION

### 2.1 Chief complaints

A 37‐year‐old woman, without a relevant medical history, noticed a lump of a size of 2 cm wide with painless and tenderness in her left breast in early 1997 but was not concerned. The breast mass progressively increased and locked her into immobility until April 2022, when it had a fever for 3 weeks, with dizziness in the eyes, pale skin, uncontrolled pain, and she was administrated in the breast surgery department of the sixth affiliated hospital of Sun Yat‐Sen university.

### 2.2 Physical examination

**FIGURE 1:**
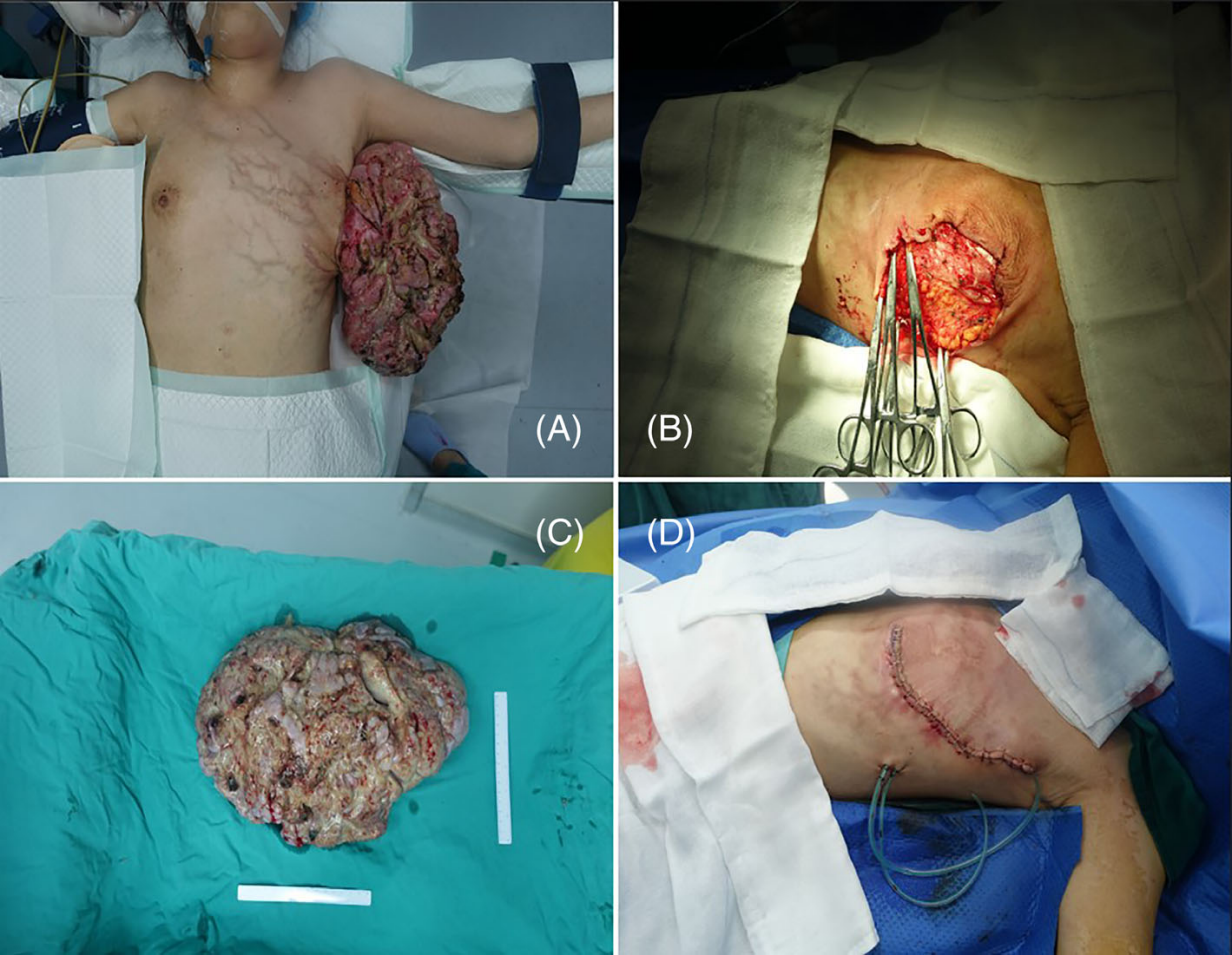
The picture of the tumor, the appearance of the tumor before surgery (Figure 1A, B), the tumor had been cut off (Figure 1C), the wound after surgery (Figure 1D).

A large mass measured of 30 × 20 × 15 cm^3^ presented ahead of left breast in macroscopic scale (shown in Figure 1). Local erosion of the squamous epithelium was observed accompanied by inflammatory exudation and ulcer formation, purulent secretions, and a small amount of bright red blood was observed on the surface, part of the ductal epithelium was detached, with inflammatory exudation and necrotic material similar to cellulose on the surface. No blood overflow was detected from bilateral papilla extrusion, enlarged lymph nodes were detected in the left axilla and the right breast was normal. The nipple was preserved on the skin inferior to the tumor. She had no history of contraceptive, personal pathology or family history of DVT, BMI was 22.15.

## 3 INVESTIGATIONS

### 3.1 Radiology

**FIGURE 2:**
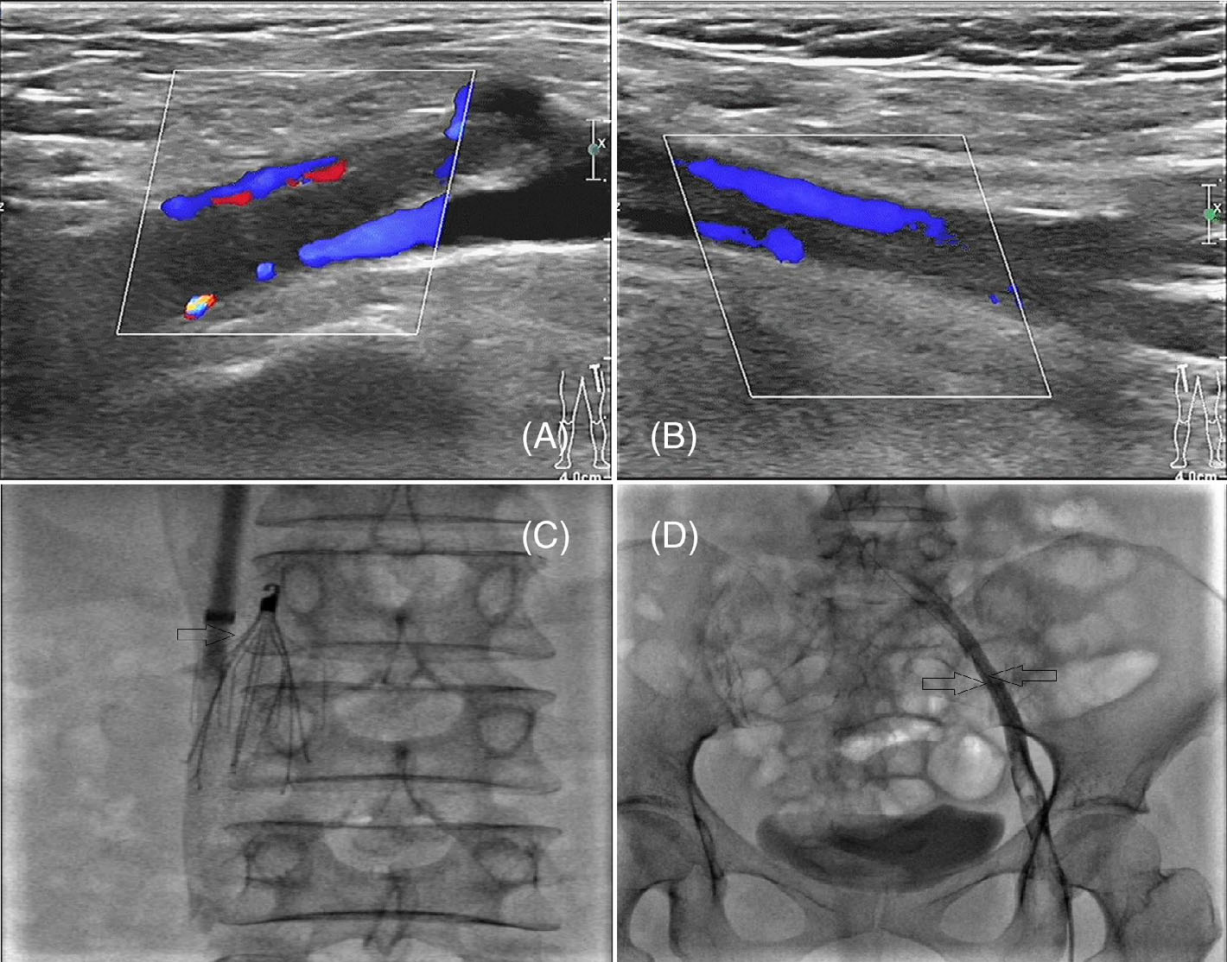
Venous thromboembolism (VTE), Sheath angiography detected local filling defects in the distal inferior vena cava and the left external femoral iliac vein (Figure 2A, B), a filter was placed in the inferior vena cava (IVC) at the thrombus site (Figure 2C, D).

Color Doppler ultrasound showed a large mass lesion in the left breast (BI‐RADS Type 4b), solid nodules in the left breast (BI‐RADS Type 3). Sheath angiography detected local filling defects in the distal inferior vena cava (IVC) and the left external femoral iliac vein, double‐limb CDU examined deep VTE (shown in Figure 2). There was no mediastinal deviation, the structure of both pulmonary hila was clear, several enlarged lymph nodes could be seen in the mediastinum, normal shape, and the short diameter of the large node was approximately 7 mm. Thyroid color ultrasound: Multiple cystic nodules in the bilateral lobes of the thyroid (ACR TI‐RADS TR1), no enlarged lymph nodes in the bilateral neck.

**FIGURE 3:**
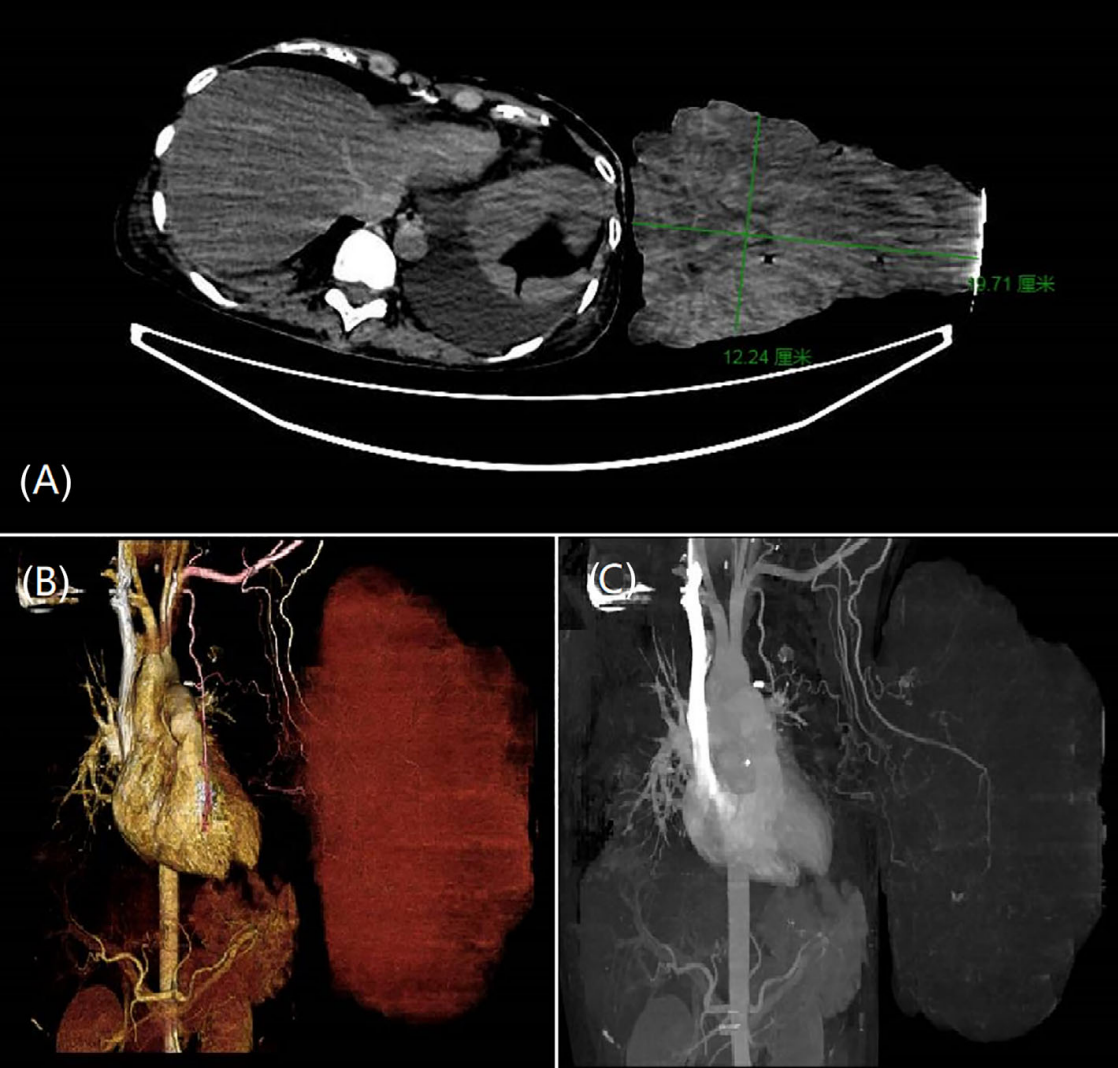
Initial computed tomography (CT) scan showing a heterogenous mass, sagittal plane (Figure 3A), transverse plane (Figure 3C), CT angiography (Figure 3B).

Computed tomography (CT) angiography presented increased vascularity within the tumor (shown in Figure 3), the normal glandular structure was difficult to distinguish due to the large irregular soft tissue mass, the lesion grew outward to the chest wall, enhanced scanning showed multiple small blood vessel shadows in the lesion, and the whole lesion showed obvious uneven enhancement. Color Doppler flow imaging did not observe a clear blood flow signal in these lesions. Positron emission tomography (PET) showed several enlarged lymph nodes in the left axilla, the enlarged lymph nodes had a short diameter of 6 mm.

## 4 BIOCHEMICAL DETECTION

### 4.1 Laboratory examinations

The basic condition of the patient was poor with severe anemia and hypoalbuminemia at admission. Blood routine found that CRP was 147 380 mg/L, RBC was 2.70010E12/L, RDW‐CV was 15 800 × 109/L, MCH was 259 000 pg, HGB was 70 000 g/L, HCT was 0.239%, LYMPHP was 0.098%, PLT was 402.00010E9/L, D dimer was 1.17 μg/mL, PT was 13.6 s. Blood leukocytes, sex hormones, and tumor markers were normal. Blood comes from the perforating branch of the left internal thoracic artery and the branch of the lateral thoracic artery.

### 4.2 Pathology

**FIGURE 4:**
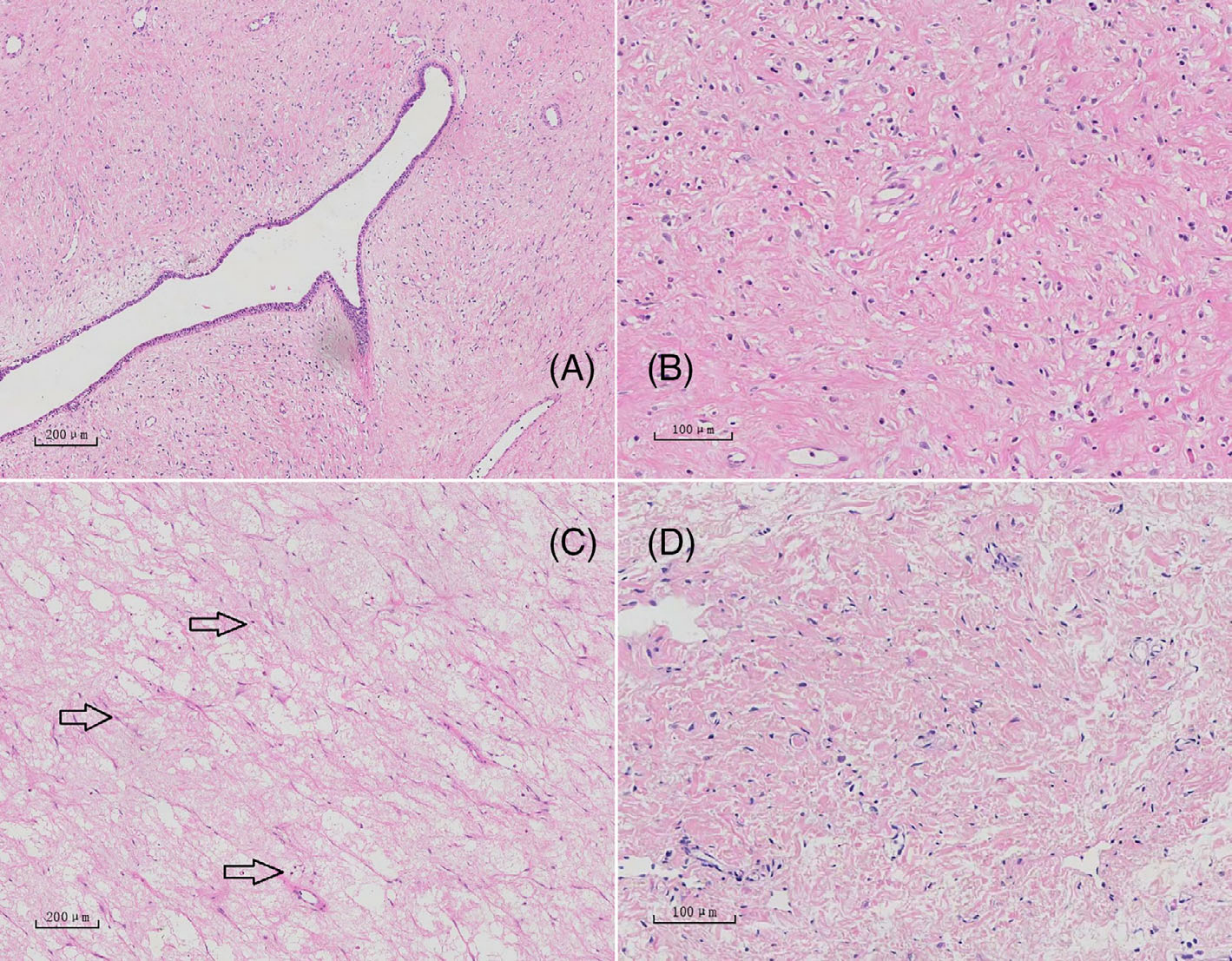
Pathology, (40×) Well‐circumscribed fibroepithelial neoplasm, prominent leaf‐like architecture and areas of hypocellular stroma (Figure 4A), (100×) Bland stromal spindle cells without mitoses or nuclear atypia (Figure 4B), (40×) low Ki‐67 expression for the stromal component, and benign character of the tumor (Figure 4C), (100×) no metastasis was observed in the left axillary lymph node (Figure 4D).

Microscopically, Hematoxylin and Eosin Staining Kit was used (60524ES60), breast tissue and striated muscle tissue were observed and no evidence of malignancy was found. The histological examination of the mass showed a negative margin which was consistent with benign PT, histological sections revealed a circumscribed lesion with a variable leaf‐like growth pattern (shown in Figure 4A). Most of the stromal component showed slight hyperplasia of the interstitial fiber tissue and muscle fiber tissue accompanied by a small amount of inflammatory cell infiltration, and absent stromal overgrowth and mitoses (shown in Figure 4B). The Ki‐67 proliferation index of the tumor was low for the stromal component (shown in Figure 4C). Left lymph nodes pathology showed no tumor metastasis (shown in Figure 4D). Immunohistochemistry found CK8/18 (+), ER (+), PR (−), Vimentin (+), ALK (−), Desmin (+), CK (+), SMA (+), CD34 (+), and low Ki‐67 proliferation index.

## 5 FINAL DIAGNOSIS

Benign PT, venous thrombosis of both lower extremities.

## 6 TREATMENT

The patient was venipunctured under ultrasound guidance, guide wire and sheath were inserted, we placed a filter (Specifications: DL950F; Batch number: GFFW2161) in the IVC at the thrombus site (shown in Figure 2C, D), an anticoagulant drug (Enoxaparin 6000AxaIU once a day plus Rivaroxaban 15 mg twice a day) of 4 weeks was used for thrombolytic therapy after the operation, swelling and pain of both lower extremities were relieved, and index‐related thrombus tended to be normal at reexamination. Then, we operated “radical resection of the left mastectomy + left axillary lymph node dissection (ALND) + partial excision of the pectoralis major + formation of a pedicled composite flap + negative pressure‐assisted healing “on the patient. Patient was given 1.5 g dosage of cefuroxime half an hour before the surgery, a 6 cm arc incision was made beside the left breast areola to cut the skin and separate the subcutaneous tissue. A large external convex mass, 30 × 20 cm^2^ in size, was completely resected at 3 o'clock of the left breast. All resection margins were free of neoplastic tissue. The mass was slightly hard in texture, with clear boundaries, gray in section and coarse calcification was observed inside, the tumor in the mastectomy specimen measured 30 × 20 × 15 cm^3^. The patientreceived ongoing anticoagulant therapy (rivaroxaban 20 mg once a day) and anti‐infection (cefuroxime 750 mg, q8h) for 5 days, resumed diet after 6 h of the operation without radiation therapy or chemotherapy due to wide excision, low expression of Ki‐67, and benign tumor characteristics.

## 7 OUTCOMES AND FOLLOW‐UP

The patient was discharged 4 days after the operation and treated with continuous anticoagulation drug for 6 months (Rivaroxaban 20 mg once a day). At the first month of discharged follow‐up, the wound healed well without skin grafting required for closure, at the sixth month of discharge, the patient made a good recovery and the mammography showed no evidence of recurrence.

## 8 DISCUSSION

Venous thromboembolism is a leading cause of perioperative morbidity and mortality,^2^ the incidence ratio of VTE in breast cancer patients is approximately 1.2%,^4^ while the incidence of VTE in patients with benign indications is relatively rare with the incidence ratio of 0.2%–0.6%.^5^, ^6^ This case presents a VTE and malignant character, was eventually pathologically confirmed to be benign PT. This finding describes the atypical features of giant benign PT and reminds the surgeon to consider the silent thromboembolism and risk when encountering a massive breast ulcerative mass and prevent serious consequences caused by missed diagnosis. It's also important to identify whether the tumor is benign or malignant when encounter giant breast PT, and avoid excessive treatment.

The concept of tumors related to venous thrombosis was proposed In 1823 for the first time, it is generally believed that VET is one of the high risk factors for malignant tumors, and VET is a common and serious complication of malignant tumors, and they are closely related.^7^, ^8^ CAT is generally produced by cancer cells and plays an important role in fibrin synthesis and platelet activation.^9^ In this case, both CDU images and blood test results showed DVT in double lower limbs, which mainly associated with the local erosion of squamous epithelium of giant lobular mass, both purulent rupture and tumor cells released a large number of inflammatory cytokines, which activated coagulation procedure and induced venous thrombosis, increased tissue damage and decreased levels of Antithrombin III may contribute to coagulopathy.^10^ The patient was treated with an IVC filter prior to surgery and underwent continuous anticoagulant therapy postoperatively.

The typical appearance of a PT on mammography have a smooth, multinodular, well‐defined, firm mass that is mobile and painless. Shiny, stretched, and attenuated skin may be seen overlying a large tumor.^11^, ^12^, ^13^ The tumor described here performed with ulceration, purulent secretions, bleeding, with inflammatory and necrotic exudation on the surface which make the case particular.

According to NCCN guidelines, chemotherapy or endocrine therapy is not supported in mastectomy patients unless there is a positive surgical margin.^14^ The standard of treatment in PTs remains the surgical approach. Ziteng Liu, Zongyan Li reported a case of benign giant PT which also presented with malignant character initially, they performed mastectomy of the tumor and did not treat with chemotherapy and endocrine therapy.^15^ Ramona‐Andreea Matei reported a left mastectomy of a 24‐year‐old woman who presented a rapid increase in volume of the left breast which was approved to be PTs.^16^ We completely resected the large external convex mass at 3 o'clock of the left breast, although axillary metastases of PT are rare, we did the left ALND due to the PET/CT report, mammography was reviewed after 6 months and no recurrence at the surgery site. We successfully removed 3.85 kg of benign PT and placed an IVC filter in the postcaval vein before surgery and performed post‐process thrombolytic therapy, this report provides evidence of the selection of thrombolytic therapy for deep vein thrombosis of the lower extremities in the setting of benign breast PTs, which can guide other surgeons in treating a similar condition.

## AUTHOR CONTRIBUTIONS

Qinbo Wang contributed to the conception of the report. Lina Wei contributed to the acquisition of clinical pictures. Haiyan Li contributed to the conception and design of the case report. Qinbo Wang, Lina Wei, Yuan Zhou, Yingjuan Ou, and Haiyan Li contributed to the drafting of the manuscript, critical reversion, and approval of the final version of the manuscript.

## CONFLICT OF INTEREST STATEMENT

The authors have stated explicitly that there are no conflicts of interest in connection with this article.

## ETHICS STATEMENT

Written informed consent was obtained from the patient for the publication of this case report and any accompanying images. This study protocol was reviewed and approved by the Ethics Committee of the Sixth Affiliated Hospital of Sun Yat‐sen University, approval number 2021ZSLYEC‐404. Supported by National Key Clinical Discipline.

## Data Availability

All data generated or analyzed during this study are included in this article. Further inquiries can be directed to the corresponding author. Data openly available in a public repository.
